# Obesity correlates with the immunosuppressive ILC2s‐MDSCs axis in advanced breast cancer

**DOI:** 10.1002/iid3.1196

**Published:** 2024-03-19

**Authors:** Wei Liu, Bingyu Li, Dan Liu, Bing Zhao, Gang Sun, Jianbing Ding

**Affiliations:** ^1^ School of Public Health Xinjiang Medical University Urumqi People's Republic of China; ^2^ Department of Mammary Medicine Affiliated Tumor Hospital of Xinjiang Medical University Urumqi People's Republic of China; ^3^ Department of Internal Medicine the Third Clinical College of Xinjiang Medical University Urumqi People's Republic of China; ^4^ Xinjiang Uygur Autonomous Region Cancer Center/Xinjiang Key Laboratory of Oncology Urumqi People's Republic of China; ^5^ Xinjiang Key Laboratory of Molecular Biology for Endemic Diseases Urumqi Xinjiang People's Republic of China; ^6^ Key Laboratory of Oncology of Xinjiang Uyghur Autonomous Region Urumqi Xinjiang People's Republic of China; ^7^ Department of Breast and Thyroid Surgery Affiliated Tumor Hospital of Xinjiang Medical University Urumqi Xinjiang People's Republic of China; ^8^ Department of Immunology, School of Basic Medical Sciences Xinjiang Medical University Urumqi Xinjiang People's Republic of China

**Keywords:** advanced breast cancer, group 2 innate lymphoid cells, inflammatory factors, interleukin, myeloid‐derived suppressor cells, obesity

## Abstract

**Aim:**

We investigated the relationship between the group 2 innate lymphoid cells (ILC2s)‐myeloid‐derived suppressor cells (MDSCs) axis and obesity‐related breast cancer.

**Methods:**

Fifty‐eight patients with breast cancer who had first relapse and metastasis between January 2019 and August 2021 were enrolled. The proportions of ILC2s and MDSCs in blood and the levels of cytokines in serum were detected with flow cytometry. Correlation analysis among clinical characteristics (such as body mass index [BMI]), cytokines, ILC2s, and MDSCs was conducted.

**Results:**

There was a significant difference in the proportions of ILC2s and MDSCs between the high BMI group and the normal BMI group (*p* < .05). In the triple‐negative breast cancer (TNBC) patients, the proportions of ILC2s and MDSCs in the obese group were significantly higher than those in the nonobese group (*p* < .05). In all breast cancer patients, there was a positive correlation between BMI and the ILC2s‐MDSCs axis (*p* < .05). However, there was no correlation observed between the number of metastases, progression‐free survival, and the ILC2s‐MDSCs axis (*p* > .05). Additionally, ILC2s showed positive correlations with MDSCs, interleukin‐5 (IL‐5), IL‐10, IL‐17A, (PD‐L1), programmed cell death 2 ligand 2 (PD‐L2), and molecular typing (*p* < .05). Similarly, MDSCs exhibited positive correlations with IL‐5, IL‐8, IL‐9, IL‐17A, PD‐L1, and PD‐L2 (*p* < .05). In patients with TNBC, there was a positive correlation between BMI and IL‐5 (*p* < .05).

**Conclusion:**

Conclusively, obesity may enhance the immunosuppressive effect of the ILC2‐MDSC axis in advanced breast cancer. IL‐5 may play a vital role in the ILC2‐MDSC axis and obesity in TNBC.

## Graphical abstract

Our findings demonstrate that the group 2 innate lymphoid cells (ILC2s)‐myeloid‐derived suppressor cells (MDSCs) axis is associated with the unfavorable prognosis of advanced breast cancer in obese patients, and cytokine interleukin‐5 (IL‐5) may be involved in this process. This study may provide experimental evidence for revealing the mechanism underlying the poor prognosis of obesity‐related breast cancer, and for developing biomarkers and clinical therapeutic targets for obesity‐related breast cancer.

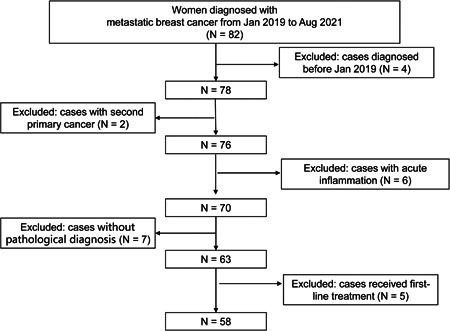

## 1 INTRODUCTION

Breast cancer has become the malignant tumor with the highest incidence worldwide,^1^ and is also the leading cause of death of women with cancer in most countries.^2^ Obesity is associated with poor prognosis of breast cancer.^3^ The tumor stage at initial diagnosis of obese women with breast cancer is high.^4^ Obesity is also related to the poorer quality of life of breast cancer patients^5^ and makes breast cancer more prone to lung metastasis.^6^ Among women with a body mass index (BMI) greater than 40, obesity significantly increases the risk of recurrence and death of breast cancer.^3^ Bandera et al.^7^ showed that central obesity and more fat would increase the risk of breast cancer in black women. Obesity may promote the recruitment of inflammatory cytokines, interference with immune surveillance, and tumor immune escape,^8^, ^9^ thus mediating its effects in breast cancer. However, the exact mechanism has not been fully elucidated.

Group 2 innate lymphoid cells (ILC2s) play a crucial role in the obesity‐related inflammatory response, contributing to the maintenance of white adipose tissue (WAT) homeostasis.^10^ Macrophages, which exhibit both anti‐inflammatory and proinflammatory effects, also play a pivotal role in maintaining WAT homeostasis. ILC2‐derived interleukin‐4 (IL‐4) and IL‐13 stimulate M2‐like macrophages, resulting in their anti‐inflammatory effects.^11^ Obesity disrupts the role of ILC2s in WAT homeostasis, leading to the polarization of M2 macrophages toward M1, which exhibits proinflammatory effects.^12^ ILC2s are also increased in a variety of malignant tumors. They can promote tumor growth by activating Treg cells and myeloid‐derived suppressor cells (MDSCs) and induce drug resistance of immune checkpoint inhibitors by promoting the expression of PD‐1.^13^, ^14^ MDSCs have a strong immunosuppressive function. In cancer, MDSCs are elevated under the stimulation of various inflammatory factors and can promote tumor progression and metastasis.^15^, ^16^ The accumulation of lipids can increase the inhibitory activity of MDSCs.^15^ Jou et al.^17^ showed that both ILC2s and MDSCs were increased in human and mouse models of colon cancer, and ILC2s promoted the progression of colon cancer by enhancing the immunosuppressive effect of MDSCs. However, the mechanism of ILC2s‐MDSCs in obesity‐related breast cancer is not clear. Therefore, we hypothesize that ILC2s may enhance the immunosuppressive effects of MDSCs through obesity mediated inflammatory environment, accelerate metastasis and drug resistance, and induce a worse prognosis of obesity.

This study aims to investigate the relationship between ILC2s‐MDSCs and obesity‐related breast cancer in clinical practice. Our findings may shed light on the immunological mechanism of relapse, metastasis, and drug resistance of obesity‐related breast cancer.

## 2 MATERIALS AND METHODS

### 2.1 Study design and participants

This is a prospective case–control study without intervention. Breast cancer patients who were hospitalized in Xinjiang Medical University Cancer Hospital from January 2019 to August 2021 were recruited. Inclusion criteria: (1) patients with advanced breast cancer diagnosed for the first time; (2) patients who did not receive treatment for advanced breast cancer; (3) patients who were diagnosed with invasive breast cancer by pathology and immunohistochemistry; Exclusion criteria: (1) patients with recurrent or metastatic breast cancer but not for the first time; (2) patients who had received treatment for advanced breast cancer. For healthy control (*n* = 20), female healthy volunteers who received physical examination during the same time were enrolled. Fasting blood was collected from each participant. This study was approved by the Ethics Committee of Affiliated Tumor Hospital of Xinjiang Medical University (Ethics No.: G‐2019016). All participants signed the informed consent.

### 2.2 Follow‐up

All patients were followed up through medical records or telephone calls. The follow‐up ended in August 2023. The progression‐free survival (PFS) was defined as the time from the beginning of first‐line treatment to the first disease progression or death, or to the latest follow‐up or data collection deadline (August 2023). The overall survival (OS) was defined as the time from the first diagnosis or recurrence to death (any reason), or to the latest follow‐up.

### 2.3 Data collection

The clinical data, such as age, BMI, Ki‐67, androgen receptor, P53, molecular typing, and metastasis number, were collected from all patients. The data on age and BMI of all patients at the time of the first metastasis were collected. When there was immunohistochemical data of the primary and metastatic breast cancer, the immunohistochemical data of the metastatic breast cancer was collected. According to the consensus of Chinese experts on nutritional medical treatment for overweight/obesity in 2016, BMI ≥ 28 kg/m^2^ was defined as obesity, 24 kg/m^2^ ≥ BMI ＜28 kg/m^2^ as overweight, and, 18.5 kg/m^2^ ≥ BMI ＜24 kg/m^2^ as normal weight. Based on BMI, patients were divided into the high BMI group (obesity and overweight) and the normal BMI group.

### 2.4 Cytometric bead assay

Cytokine levels in serum were detected with cytometric bead assay and cytokine detection kits (Biolegend), according to the kit instructions. The samples were analyzed with a DXflex flow cytometer (Beckman). The cytokine level was calculated according to the standard curve.

### 2.5 Flow cytometry analysis of ILC2 and MDSC

The blood samples were incubated with the corresponding antibodies (BD) in the dark at room temperature for 15–30 min. For ILC2, the antibodies included Lin (CD3, CD4, CD8, CD14, CD15, CD16, CD19, CD20, CD33, CD34, CD23c, and FcεRIα), CD45, CD127, and, CRTH2. For MDSC, the antibodies of CD14, CD33, CD11b, and human leukocyte antigen‐DR (HLA‐DR) were used. Then, the samples were incubated with red blood cell lysis buffer at room temperature for 8–12 min and centrifuged for 5 min. The precipitate was collected, resuspended with phosphate‐buffered saline (PBS), and analyzed on a DXflex flow cytometer (Beckman). The CD45+lin‐CD127 + CRTH2+ cells were defined as ILC2. The CD14‐HLA‐DR‐CD11b + CD33+ cells were defined as MDSC.

### 2.6 Statistical analysis

Measurement data of normal distribution are mean ± SD and were analyzed with a *t* test. Measurement data of nonnormal distribution are described by the median and interquartile range and were compared by the Wilcoxon rank sum test. The qualitative data are expressed as absolute numbers and constituent ratios, and the differences were compared by *χ* ^2^ test, Fisher's exact probability method, or corrected *χ* ^2^ test. The correlation among ILC2, MDSCs, and cytokines was analyzed with Spearman correlation analysis. The associations of ILC2 and MDSCs with clinical characteristics (BMI, molecular typing, androgen receptor, P53, and number of metastases) were evaluated with Kendall rank correlation. The two‐sided *p* value less than .05 was considered significant. SAS JMP10.0 and GraphPad Prism v8.0 were used for statistical analysis.

## 3 RESULTS

### 3.1 Baseline information of study participants

The flowchart of patient enrollment is shown in Figure S1. A total of 58 patients with advanced breast cancer, with a median age of 55.5 (49, 63.5) years, were enrolled in the study. Their clinical data are listed in Table S1. There were 26 cases of hormone receptor‐positive (HR+) type, six cases of human epidermal growth factor receptor‐positive (HER2+) type, and 26 cases of triple‐negative breast cancer (TNBC) type. There were 22 cases of Han and 36 cases of minority nationality (including Uygur, Kazak, Hui, and others). There were 24 cases of obesity, 23 cases of overweight, and 11 cases of normal weight. The differences in age and ethnicity between the normal BMI group (*n* = 11) and high BMI group (*n* = 47) were not significant (*p* > .05). For healthy control, their average age was 52 ± 4 years, and their BMI was within the normal range.

### 3.2 Comparison of ILC2s, MDSCs, and cytokines

Flow cytometry was used to detect the proportions of ILC2s and MDSCs in blood. The gating strategies for ILC2s and MDSCs are shown in Figures S2A and S3A, respectively. The representative images of the proportions of ILC2s and MDSCs in different BMI groups of all patients (Figures S2B and S3B) and in obese and nonobese patients with TNBC (Figures S2C and S3C) are displayed. As shown in Table 1, there were significant differences in the proportions of ILC2s and MDSCs between normal BMI and high BMI groups (*p* < .05). PD‐L2 level in the high BMI group was significantly higher than that in the normal BMI group (*p* < .05). However, no significant differences were found in interleukin‐4 (IL‐4), IL‐5, IL‐6, IL‐8, IL‐10, and IL‐17A (*p* > .05). Furthermore, we compared the ILC2s and MDSCs of obese and nonobese patients in TNBC patients. The results showed that the proportions of ILC2s and MDSCs in the obesity group were significantly higher than those in the nonobesity group (*p* < .05) (Table 2).

**Table 1:** Comparison of ILC2s, MDSCs, and cytokines between normal and high BMI groups of all patients (M (Q25, Q75)).

	Normal BMI group (*n* = 11)	High BMI group (*n* = 47)	*Z*	*P*
ILC2s (%)	0.77(0.25,0.98)	1.42(0.98,2.24)	3.392	**<.001**
MDSCs (%)	0.54(0.32,0.93)	1.08(0.80,1.97)	3.809	**<.001**
IL‐5 (pg/mL)	31.09(24.08,45.26)	33.89(21.92,44.47)	0.268	.789
IL‐2 (pg/mL)	3.61(2.02,4.01)	3.26(1.78,4.20)	0.337	.736
IL‐4 (pg/mL)	9.25(6.43,23.83)	10.65(4.92,17.19)	0.436	.663
IL‐6 (pg/mL)	19.48(11.79,29.51)	23.57(12.42,30.11)	0.754	.451
IL‐8 (pg/mL)	26.39(24.56,31.41)	26.49(21.37,28.93)	1.250	.212
IL‐9 (pg/mL)	3.98(1.52,8.71)	3.13(1.87,6.08)	0.635	.526
IL‐10 (pg/mL)	4.27(3.69,6.54)	4.67(3.32,6.35)	0.119	.905
IL‐13 (pg/mL)	8.35(3.69,10.45)	4.05(2.74,12.08)	0.615	.539
IL‐22 (pg/mL)	6.13(4.57,23.95)	5.07(3.27,10.48)	1.289	.197
IL‐25 (pg/mL)	1.04(0.87,1.39)	1.13(1.01,1.34)	0.823	.410
IL‐33 (pg/mL)	2.53(2.17,2.76)	2.44(2.19,2.75)	0.268	.789
PD‐L1 (pg/mL)	35.61(33.11,41.75)	42.42(34.38,48.05)	1.745	.081
PD‐L2 (pg/mL)	32.17(28.94,35.83)	37.87(33.21,45.79)	2.241	**.025**
PD‐1 (pg/mL)	3.87(2.86,4.51)	3.51(2.36,4.17)	0.724	.469
IFN‐gamma (pg/mL)	34.37(22.98,58.17)	35.41(23.75,40.27)	0.575	.565
TNF‐γ (pg/mL)	11.68(3.71,23.3)	14.15(6.67,23.45)	0.754	.451
IL‐17A (pg/mL)	1.75(1.16,3.73)	2.35(1.58,5.46)	1.121	.262

*Note*: Bold values indicate statistically significant differences at *P* < .05.

Abbreviations: BMI, body mass index; IFN‐γ, interferon‐γ; ILC2s, group 2 innate lymphoid cells; MDSCs, myeloid‐derived suppressor cells; PD‐L1, programmed cell death 1 ligand 1; PD‐L2, programmed cell death 2 ligand 2; PD‐1, programmed cell death 1; TNF‐α, tumor necrosis factor‐α.

**Table 2:** Comparison of ILC2s and MDSCs between non‐obesity and obesity groups of TNBC patients (M (Q25, Q75) or mean ± SD).

	Nonobesity group (*n* = 13)	Obesity group (*n* = 13)	*Z*/*t*	*P*
ILC2s (%)	1.02 ± 0.62	2.67 ± 1.04	4.920	**<.001**
MDSCs (%)	0.86 (0.59, 0.95)	1.78 (1.11, 2.75)	3.847	**<.001**

*Note*: Bold values indicate statistically significant differences at *P* < .05.

Abbreviations: ILC2s, group 2 innate lymphoid cells; MDSCs, myeloid‐derived suppressor cells; TNBC, triple‐negative breast cancer.

### 3.3 Correlation analysis of clinical characteristics, cytokines, ILC2s, and MDSCs

**Figure 1:**
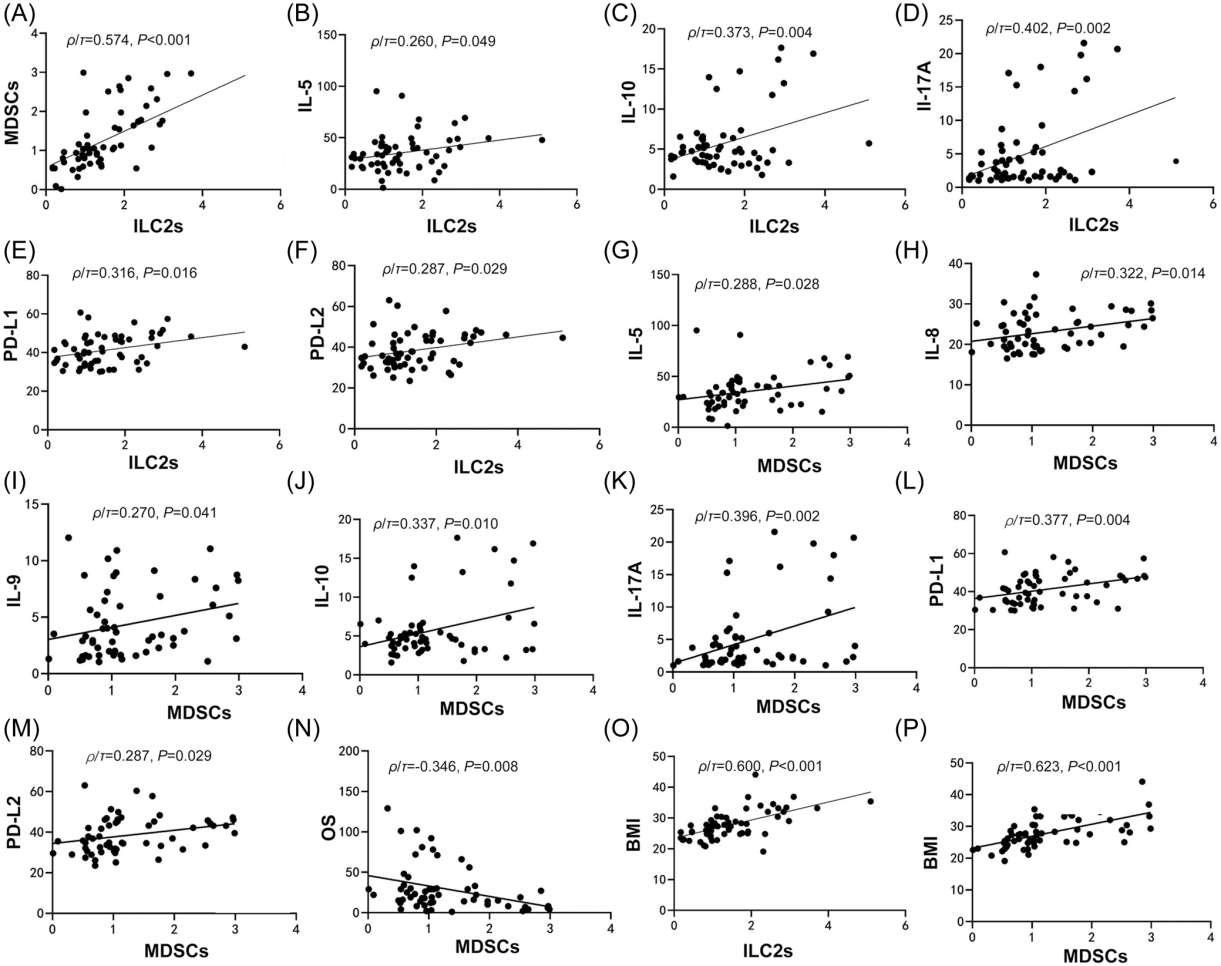
Correlation analysis. (A) Correlation of group 2 innate lymphoid cells (ILC2s) with myeloid‐derived suppressor cells (MDSCs). (B) Correlation of ILC2s with interleukin 5 (IL‐5). (C) Correlation of ILC2s with IL‐10. (D) Correlation of ILC2s with IL‐17A. (E) Correlation of ILC2s with programmed cell death 1 (PD‐L1). (F) Correlation of ILC2s with programmed cell death 2 ligand 2 (PD‐L2). (G) Correlation of MDSCs with IL‐5. (H) Correlation of MDSCs with IL‐8. (I) Correlation of MDSCs with IL‐9. (J) Correlation of MDSCs with IL‐10. (K) Correlation of MDSCs with IL‐17A. (L) Correlation of MDSCs with PD‐L1. (M) Correlation of MDSCs with PD‐L2. (N) Correlation of MDSCs with overall survival (OS). (O) Correlation of ILC2s with body mass index (BMI). (P) Correlation of MDSCs with BMI.

The assignment values of BMI, molecular typing, Ki‐67, androgen receptor, P53, metastasis number, PFS, and OS are shown in Table S2. The correlation analysis results demonstrated significant positive correlations between ILC2s and MDSCs (Figure 1A), IL‐5 (Figure 1B), IL‐10 (Figure 1C), IL‐17A (Figure 1D), PD‐L1 (Figure 1E), and PD‐L2 (Figure 1F) (*p* < .05) (Table 3). MDSCs also exhibited positive correlations with IL‐5 (Figure 1G), IL‐8 (Figure 1H), IL‐9 (Figure 1I), IL‐10 (Figure 1J), IL‐17A (Figure 1K), PD‐L1 (Figure 1L), and PD‐L2 (Figure 1M), but a negative correlation with OS (Figure 1N) (*p* < .05). Additionally, BMI displayed positive correlations with ILC2s (*ρ/r* = .600, *p* < .001, Figure 1O) and MDSCs (*ρ/r* = .623, *p* < .001, Figure 1P) (*p* < .05).

**Table 3:** Correlation analysis.

	ILC2s (%)		MDSCs (%)	
	*ρ/r*	*P*	*ρ/r*	*P*
BMI	0.600	**<.001**	0.623	**<.001**
Ki‐67	0.089	.505	0.035	.793
Androgen receptor	−0.135	.370	−0.077	.613
P53	−0.163	.286	−0.089	.561
Molecular typing	0.267	**.044**	0.189	.156
Metastasis number	−0.190	.153	0.089	.508
IL‐5 (pg/mL)	0.260	**.049**	0.288	.028
IL‐2 (pg/mL)	0.152	.256	0.034	.800
IL‐4 (pg/mL)	0.191	.150	0.154	.250
IL‐6 (pg/mL)	0.158	.236	0.207	.118
IL‐8 (pg/mL)	0.187	.161	0.322	**.014**
IL‐9 (pg/mL)	0.153	.252	0.270	**.041**
IL‐10 (pg/mL)	0.373	**.004**	0.337	**.010**
IL‐13 (pg/mL)	−0.036	.786	−0.014	.918
IL‐25 (pg/mL)	0.155	.246	0.054	.686
IL‐33 (pg/mL)	0.084	.529	0.139	.298
PD‐L1 (pg/mL)	0.316	**.016**	0.377	**.004**
PD‐L2 (pg/mL)	0.287	**.029**	0.287	**.029**
PD‐1 (pg/mL)	−0.129	.336	−0.221	.095
IL‐17A (pg/mL)	0.402	**.002**	0.396	**.002**
PFS (month)	0.110	.413	−0.238	.074
OS (month)	−0.028	.834	−0.346	**.008**
MDSCs (%)	0.574	**<.001**	‐	‐

*Note*: Bold values indicate statistically significant differences at *P* < .05.

Abbreviations: BMI, body mass index; ILC2s, group 2 innate lymphoid cells; MDSCs, myeloid‐derived suppressor cells; OS, overall survival; PD‐L1, programmed cell death 1 ligand 1; PD‐L2, programmed cell death 2 ligand 2; PD‐1, programmed cell death 1; PFS, progression‐free survival.

### 3.4 Correlation analysis between BMI and cytokines

**Figure 2:**
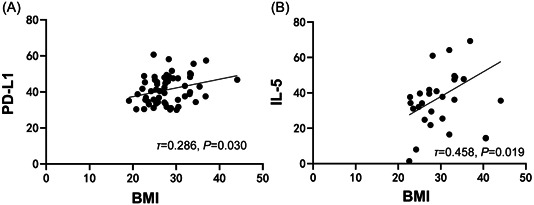
Correlation analysis of body mass index (BMI) with programmed cell death 1 (PD‐L1) and interleukin 5 (IL‐5). (A) Correlation of BMI with PD‐L1 in all patients. (B) Correlation of BMI with IL‐5 in triple‐negative breast cancer (TNBC) patients.

We found that BMI was positively correlated with PD‐L1 in all patients (*p* < .05) (*τ* = 0.286, *p* = .030, Figure 2A). In patients with TNBC, there was also a positive correlation between BMI and IL‐5 (*p* < .05) (*r* = .458, *p* = .019, Figure 2B).

### 3.5 Comparison of cytokines among healthy control, normal BMI group, and high BMI group

**Figure 3:**
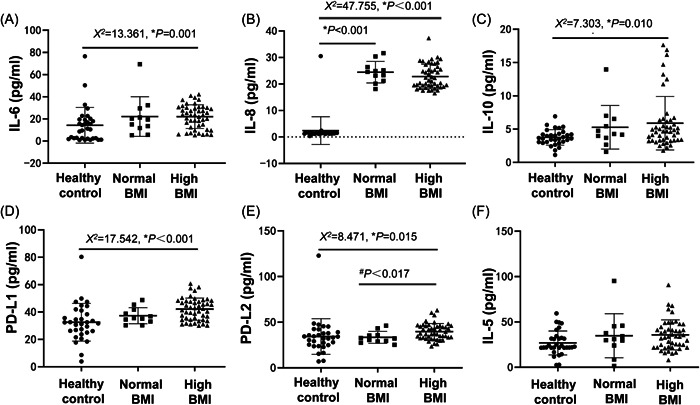
Comparison of cytokines among healthy control, normal body mass index (BMI) group, and high BMI group. The levels of interleukin 6 (IL‐6) (A), IL‐8 (B), IL‐10 (C), programmed cell death 1 (PD‐L1) (D), programmed cell death 2 ligand 2 (PD‐L2) (E), and IL‐5 (F) were compared among healthy control, normal BMI group, and high BMI group.

We further compared the cytokines among the healthy control, normal BMI group, and high BMI group. The results showed that the levels of IL‐6 (Figure 3A), IL‐8 (Figure 3B), IL‐10 (Figure 3C), PD‐L1 (Figure 3D), and PD‐L2 (Figure 3E) in the high BMI group were significantly higher than the normal BMI group (*p* < .05). The normal BMI group had significantly higher IL‐8 than the healthy control (*p* < .05). In addition, there was a significant difference in PD‐L2 between the normal BMI group and the high BMI group (*p* < .05). However, there was no significant difference in IL‐5 (Figure 3F) among the three groups.

## 4 DISCUSSION

ILC2s are derived from bone marrow lymphoid progenitor cells. They can mediate tumor development and affect tumor prognosis by secreting type 2 cytokines such as IL‐4, IL‐5, IL‐9, and IL‐13. They can also recruit MDSCs and promote tumor progression.^18^, ^19^ MDSCs have immunosuppressive functions and their production can be accelerated under the stimulation of IL‐6, IL‐8, and IL‐10. High‐fat diet and obesity are conducive to the differentiation of bone marrow precursor cells into MDSCs.^10^, ^11^, ^20^, ^21^ The prognosis of obesity‐related breast cancer is poor. However, the pathogenesis of obesity‐related breast cancer has not been fully understood. We suppose that the ILC2‐MDSC immunosuppressive axis may be involved in obesity‐related breast cancer.

In this study, we found that the levels of ILC2s and MDSCs in the high BMI group were prominently higher than in the normal BMI group. Additionally, the proportions of ILC2s and MDSCs in obese TNBC patients were significantly higher than those in nonobese TNBC patients. Our results are consistent with those of Rehana et al.,^22^ which demonstrated that IL‐4, IL‐5, IL‐6, IL‐8, IL‐10, and IL‐17A in the high BMI group were higher than in the normal BMI group. However, no significant differences were found.

MDSCs are related to the poor prognosis of breast cancer.^23^ Bergenfelz et al.^24^ found that compared with the healthy control group, the level of MDSCs in the peripheral blood of patients with advanced breast cancer was significantly higher. Patients with high levels of MDSCs had a higher proportion of liver metastases, bone metastases, and multiple metastases. Additionally, the PFS and OS of patients with high levels of MDSCs were shorter. To further indicate the relationship between obesity and the immunosuppressive axis of ILC2s‐MDSCs in advanced breast cancer, we conducted a correlation analysis of clinical characteristics, prognostic indicators, cytokines, ILC2s, and MDSCs. Our results found that OS was negatively correlated with MDSCs in all the breast cancer patients enrolled in this study, consistent with the study by Bergenfelz et al.^24^ Li et al.^25^ found that the higher MDSCs level indicated a later clinical stage and that MDSCs were risk factors for drug resistance and cancer progression. Palazón‐Carrión et al.^26^ also found that lower levels of MDSCs in the peripheral blood of breast cancer patients were associated with better clinical benefits after chemotherapy. However, in patients with advanced TNBC, the correlation between the level of MDSCs and the therapeutic efficacy of pamuzumab and gemcitabine was not identified.^27^ In contrast, our results showed that ILC2s had a stronger correlation with TNBC. Unfortunately, we did not find a correlation between the number of metastases, PFS, and the ILC2s‐MDSCs axis in this study.

In all enrolled patients, IL‐5, IL‐10, IL‐17A, PD‐L1, PD‐L2 and MDSCs were positively correlated with ILC2s. In addition, IL‐25 was positively correlated with ILC2s in TNBC patients. It has been shown that the increase of ILC2s depends on the stimulation of IL‐25 and IL‐33.^17^ ILC2s have the tendency to differentiate into ILC1 and ILC3, and can secrete IL‐5, IL‐9, IL‐6, IL‐10, and IL‐17.^14^ Consistently, we found that with the increase of IL‐25, ILC2s also increased correspondingly, and IL‐5 was upregulated accordingly. Studies have shown that obesity can recruit inflammatory factors such as IL‐17A and IL‐10,^11^, ^12^, ^13^ and promote the production of MDSCs. This study observed a positive correlation between BMI and both ILC2s and MDSCs in all enrolled patients, suggesting that obesity enhances the ILC2s‐MDSCs axis. Additionally, the level of MDSCs increased with elevated levels of inflammatory factors such as IL‐5, IL‐8, IL‐9, IL‐10, and IL‐17A. These inflammatory factors are derived from ILC2s or cells differentiated from ILC2s, which further verified the role of ILC2 in upregulating MDSCs, which is consistent with previous studies.^11^, ^12^, ^13^, ^28^ In contrast, Takaharu et al. reported that both ILC2s and ILC3s contributed to obesity through the action of IL‐2.^12^ In addition, this study also found that the ILC2s‐MDSCs axis was positively correlated with PD‐L1 and PD‐L2. Consistently, it has been reported that the increase of ILC2s and MDSCs further promotes the increase of PD‐L1,^29^ leading to resistance to the immune checkpoint inhibitors. Liu et al. found that MDSCs enhanced their immunosuppressive function in breast cancer by activating the PI3K/AKT signaling pathway through PD‐1/PD‐L1.^30^

To clarify the cytokines mediating the upregulation of MDSC by ILC2 in obesity‐related breast cancer, we further performed a correlation analysis between BMI and cytokines. We found that in TNBC patients, IL‐5 had a positive correlation with BMI. Similarly, it has been shown that obesity enhances breast cancer metastasis through IL‐5.^31^ Although there was an increasing trend, IL‐5 levels were not significantly different among the healthy control, high BMI, and normal BMI metastatic breast cancer groups. This may be related to the small sample size.

This study has some limitations. For example, this is a clinical study without intervention and only preliminary results were obtained. Second, the sample size was relatively small. Third, the presence of ILC2, MDSC, IL‐5, and IL‐9 in biopsied TNBC samples could not be validated due to the limited tissue quantity available for analysis. Further studies are warranted.

## 5 CONCLUSIONS

To sum up, our findings demonstrate that the ILC2s‐MDSCs axis is associated with the unfavorable prognosis of advanced breast cancer in obese patients, and cytokine IL‐5 may be involved in this process. This study may provide experimental evidence for revealing the mechanism underlying the poor prognosis of obesity‐related breast cancer, and for developing biomarkers and clinical therapeutic targets for obesity‐related breast cancer.

## AUTHOR CONTRIBUTIONS

Wei Liu performed the research. Gang Sun and Jianbing Ding designed the research study. Bingyu Li and Dan Liu contributed essential reagents or tools. Bing Zhao analyzed the data. Wei Liu, Gang Sun, and Jianbing Ding wrote the paper. All authors have read and approved the final manuscript.

## CONFLICT OF INTEREST STATEMENT

The authors declare no conflict of interest.

## ETHICS STATEMENT

This study was performed in line with the principles of the Declaration of Helsinki. Approval was granted by the Ethics Committee of Affiliated Tumor Hospital of Xinjiang Medical University (Ethics No.: G‐2019016). All participants signed the informed consent.

## Supporting information

Supplementary Figure S1. Flowchart of patient enrollment.

**Supplementary Figure S2. Flow cytometry analysis of ILC2s**. (A) Gating strategy. (B) Proportions of IC2s in normal BMI and high BMI groups of all breast cancer patients. (C) Proportions of IC2s in non‐obesity and obesity groups of TNBC patients.

**Supplementary Figure S3. Flow cytometry analysis of MDSCs**. (A) Gating strategy. (B) Proportions of MDSCs in normal BMI and high BMI groups of all breast cancer patients. (C) Proportions of MDSCs in non‐obesity and obesity groups of TNBC patients.

Supplementary Table S1. Clinical data of patients (n (%)).

Supplementary Table S2. Assignment value for each variable.

Supporting information.

## Data Availability

The datasets generated and/or analyzed during the current study are not publicly available but are available from the corresponding author on reasonable request.
