# Supplementary figures and images for: Obesity correlates with the immunosuppressive ILC2s‐MDSCs axis in advanced breast cancer
Source: Immun Inflamm Dis. 2024 Mar 19;12(3):e1196. doi: 10.1002/iid3.1196 (PMC10949396; doi:10.1002/iid3.1196)

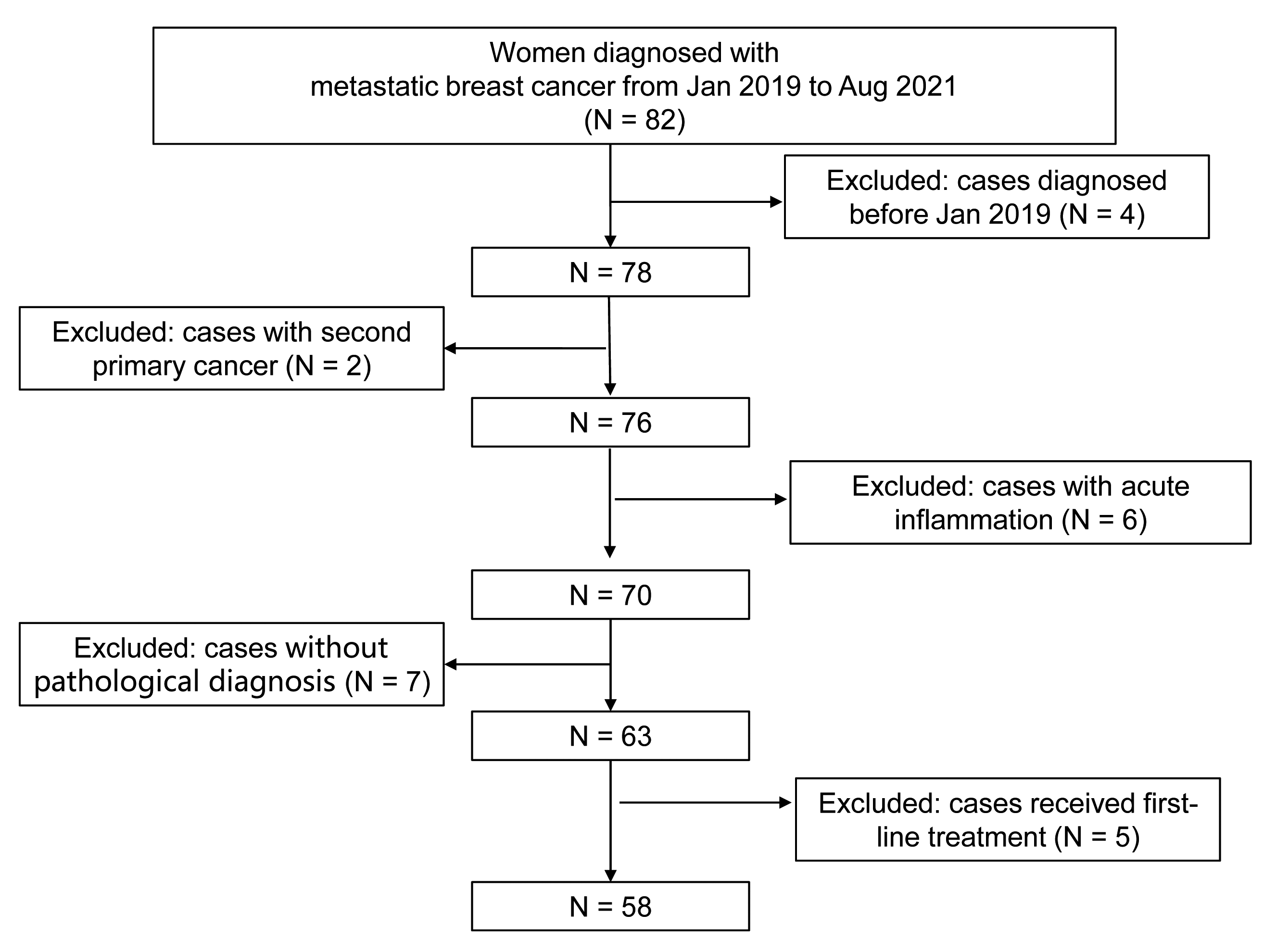

Supplement: Supplementary file 1 — Supplementary Figure S1. Flowchart of patient enrollment. [file IID3-12-e1196-s003.tif]

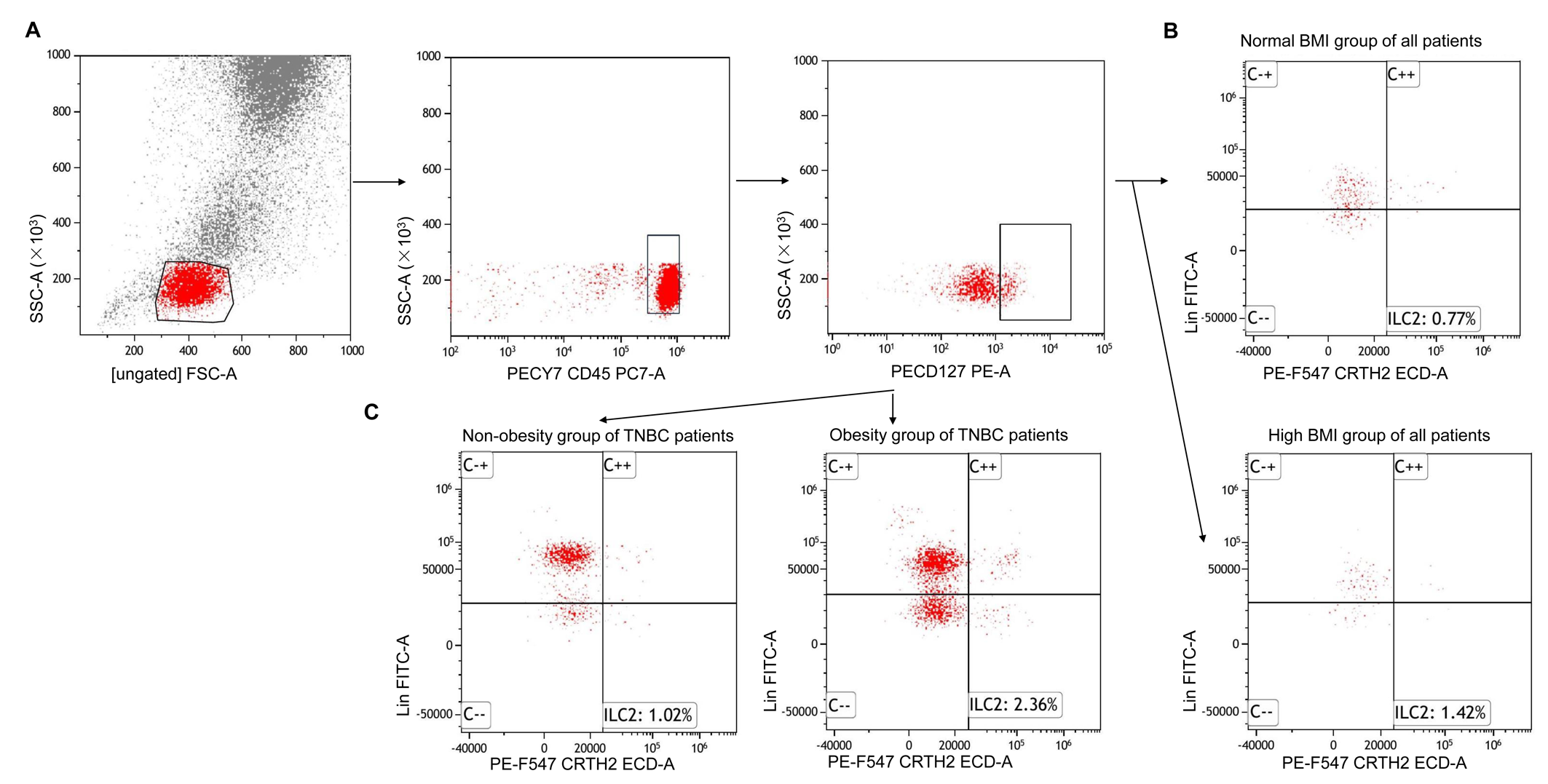

Supplement: Supplementary file 2 — Supplementary Figure S2. Flow cytometry analysis of ILC2s. (A) Gating strategy. (B) Proportions of IC2s in normal BMI and high BMI groups of all breast cancer patients. (C) Proportions of IC2s in non‐obesity and obesity groups of TNBC patients. [file IID3-12-e1196-s001.tif]

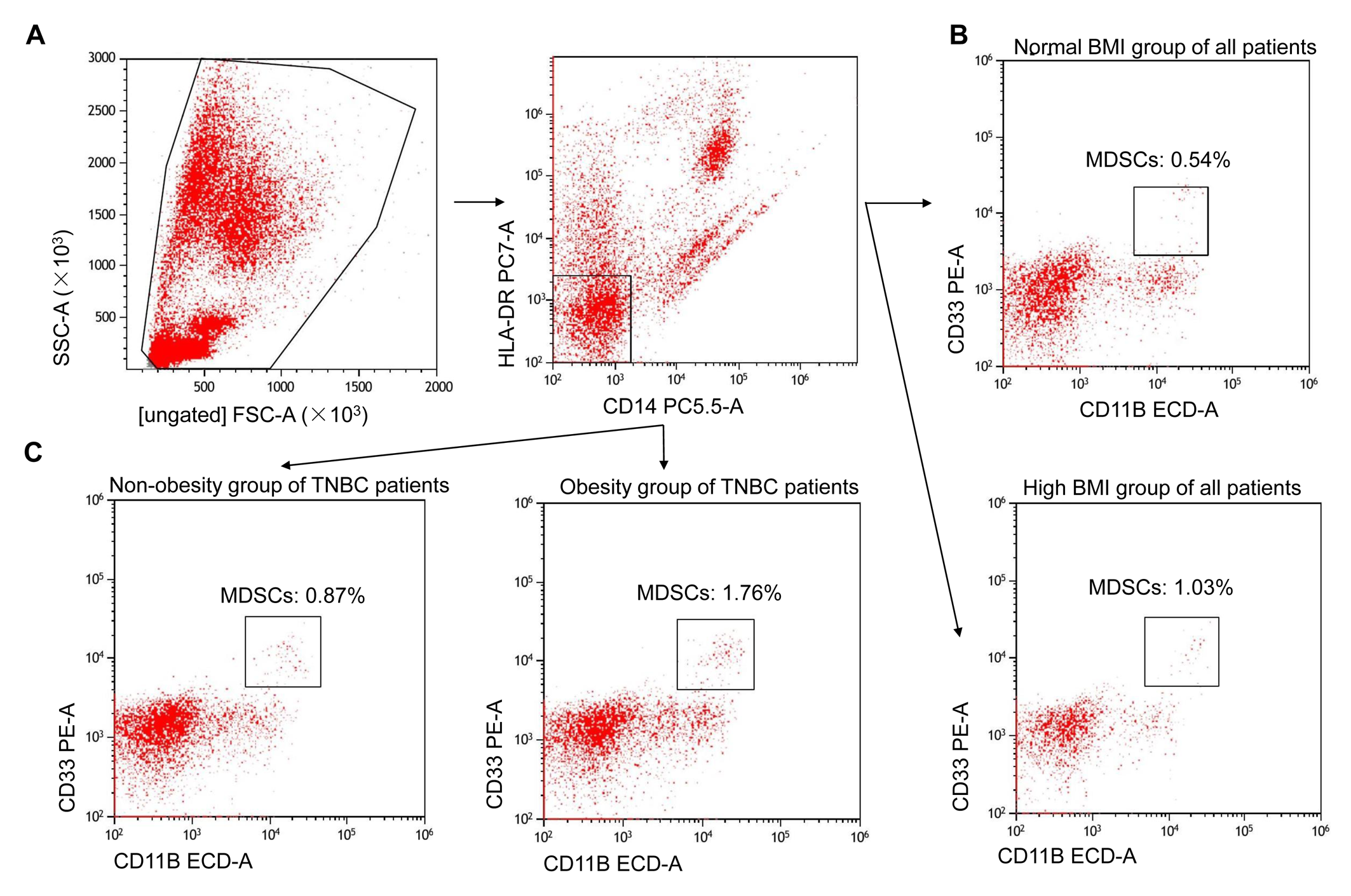

Supplement: Supplementary file 3 — Supplementary Figure S3. Flow cytometry analysis of MDSCs. (A) Gating strategy. (B) Proportions of MDSCs in normal BMI and high BMI groups of all breast cancer patients. (C) Proportions of MDSCs in non‐obesity and obesity groups of TNBC patients. [file IID3-12-e1196-s005.tif]
