# Supplementary material for: Obesity correlates with the immunosuppressive ILC2s‐MDSCs axis in advanced breast cancer
Source: Immun Inflamm Dis. 2024 Mar 19;12(3):e1196. doi: 10.1002/iid3.1196 (PMC10949396; doi:10.1002/iid3.1196)
Supplement: Supplementary file 4 — Supplementary Table S1. Clinical data of patients (n (%)). [file IID3-12-e1196-s006.docx]

**Supplementary Table S1. Clinical data of patients (n (%)).**

| Patients with advanced breast cancer | | n=58 |
| --- | --- | --- |
| Age (years) | ≤35 | 1(1.74) |
|  | 36-49 | 15(25.85) |
|  | 50-59 | 25(43.10) |
|  | ≥60 | 17(29.31) |
| Ethnicity | Han | 22(37.93) |
|  | Uygur | 23(39.66) |
|  | Kazak | 4(6.90) |
|  | Hui | 6(10.34) |
|  | Others | 3(5.17) |
| Body mass index (kg/m^2^) | 18.5-23.9 | 11(18.96) |
|  | 24.0-27.9 | 23(39.66) |
|  | ≥28 | 24(41.38) |
| Androgen receptor | Negative | 14(24.14) |
|  | Positive | 32(55.17) |
|  | None | 12(20.69) |
| P53 | Negative | 22(37.93) |
|  | Positive | 23(39.66) |
|  | None | 13(22.41) |
| Molecular typing | HR+ HER2- | 26(44.83) |
|  | TNBC | 26(44.83) |
|  | HR- HER2+ | 6(10.34) |
| Metastasis | Oligo-metastasis | 16(27.59) |
|  | Multiple-metastasis | 42(72.41) |
| Visceral metastasis | Yes | 36(62.07) |
|  | No | 22(37.93) |

**Note:** HR, hormone receptor; HER2, human epidermal growth factor receptor; TNBC, triple-negative breast cancer.
