# Supplementary material for: Obesity correlates with the immunosuppressive ILC2s‐MDSCs axis in advanced breast cancer
Source: Immun Inflamm Dis. 2024 Mar 19;12(3):e1196. doi: 10.1002/iid3.1196 (PMC10949396; doi:10.1002/iid3.1196)
Supplement: Supplementary file 5 — Supplementary Table S2. Assignment value for each variable. [file IID3-12-e1196-s004.docx]

**Supplementary Table S2. Assignment value for each variable.**

| Variable | Assignment value |
| --- | --- |
| Molecular typing | Non-TNBC type=1; TNBC type=2 |
| Androgen receptor | Negative=1; Positive=2 |
| P53 | Negative=1; Positive=2 |
| Ki-67 | <15%=1，≥15%-30%=2，≥30%=3 |
| Metastasis number | Oligometastasis=1; Multiple metastasis=2 |

Note: TNBC, triple-negative breast cancer.
